# Impact of injury duration on a sensorimotor functional network in complete spinal cord injury

**DOI:** 10.1002/jnr.25069

**Published:** 2022-05-24

**Authors:** Yun Guo, Yunxiang Ge, Jianjun Li, Weibei Dou, Yu Pan

**Affiliations:** ^1^ Department of Rehabilitation Medicine Beijing Tsinghua Changgung Hospital Beijing China; ^2^ School of Clinical Medicine Tsinghua University Beijing China; ^3^ Department of Electronic Engineering, Beijing National Research Center for Information Science and Technology (BNRist) Tsinghua University Beijing China; ^4^ School of Rehabilitation Medicine Capital Medical University Beijing China; ^5^ Department of Spinal and Neural Functional Reconstruction China Rehabilitation Research Center Beijing China

**Keywords:** brain reorganization, functional connectivity, resting‐state functional MRI, spinal cord injury

## Abstract

Connectivity changes after spinal cord injury (SCI) appear as dynamic post‐injury procedures. The present study aimed to investigate the alterations in the functional connectivity (FC) in different injury duration in complete SCI using resting‐state functional magnetic resonance imaging (fMRI). A total of 30 healthy controls (HCs) and 27 complete SCI patients were recruited in this study. A seed‐based connectivity analysis compared FC differences between HCs and SCI and among SCI subgroups (SCI patients with post‐injury within 6 months (early stage, *n* = 13) vs. those with post‐injury beyond 6 months (late stage, *n* = 14)). Compared to HCs, SCI patients showed an increase in FC between sensorimotor cortex and cognitive, visual, and auditory cortices. The FC between motor cortex and cognitive cortex increased over time after injury. The FC between sensory cortex and visual cortex increased within 6 months after SCI, while FC between the sensory cortex and auditory cortex increased beyond 6 months after injury. The FC between sensorimotor cortex and cognitive, visual, auditory regions increased in complete SCI patients. The brain FC changed dynamically, and rehabilitation might be adapted over time after SCI.

## Graphical abstract

Sensorimotor functional network in complete SCI.

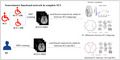

**Significance**

Spinal cord injury (SCI) disrupts the connection between the limbs and brain. It has been suggested that functional reorganization not only occurs in the local spinal cord but also in brain. Brain reorganization is not a static phenomenon but rather a dynamic process. However, few data exist on functional connectivity changes between the sensorimotor cortex and other brain regions. The present study investigated the increased sensorimotor network connectivity in complete SCI, and indicated that connectivity changed in post‐injury procedures.

## 1 INTRODUCTION

Traumatic spinal cord injury (SCI) results in catastrophic dysfunction of motor, sensory, and autonomic systems, leading to implications on physical health and psychosocial issues (Ahuja et al., 2017; Azzarito et al., 2020; Dietz & Curt 2006). Although structural recovery in the spinal cord is limited for complete SCI patients, plastic changes or functional reorganization occur in different neural circuits in the spinal cord and higher‐order structures, including subcortical and cortical structures, following SCI (Nardone et al., 2013). These neuroimaging information might guide us develop new techniques to improve the functional recovery in the limbs.

Brain reorganization is a complex process in combination with adaptive and maladaptive pathological changes after SCI (Karunakaran et al., 2020; Moxon et al., 2014). Some plastic changes are beneficial to functional recovery or prediction of clinical outcomes (Cramer et al., 2005; Hou et al., 2016; Zhu et al., 2015), while some alterations are not suitable for clinical consequences, such as neuropathic pain (Jutzeler et al., 2015, 2016; Osinski et al., 2020; Yoon et al., 2013). Therefore, optimizing cost‐effective therapies to maximize functional recovery while minimizing maladaptive states after SCI is essential (Moxon et al., 2014).

Functional connectivity (FC) is defined as statistical dependency among remote neurophysiological events (Friston, 2011). It is used as a resting‐state property in resting‐state functional magnetic resonance imaging (fMRI) studies to measure brain functional reorganization. Previous functional studies have reported decreased or increased FC between cortical sensorimotor regions following SCI (Hou et al., 2014; Karunakaran et al., 2020; Oni‐Orisan et al., 2016; Wang et al., 2019), and these FC alterations might provide significant implications for the prognosis of SCI. The increased FC between the primary motor cortex and higher‐order secondary motor areas plays a vital role in the motor recovery of SCI at 6 months post‐injury (Hou et al., 2016). Therefore, brain FC changes might be optimal clinical biomarkers to gain information about functional recovery in SCI individuals.

Although a blind person has better tactile and auditory functions to compensate for the loss of vision (Kupers & Ptito, 2014), how do other systems or regions in the brain react to the sensorimotor dysfunction in patients with SCI? Some functional studies reported FC changes between cortical sensorimotor regions after SCI (Hawasli et al., 2018), while a few studies focused on increased FC or functional changes in other brain regions, such as the visual cortex, which is crucial for movements (Scott, 2004). Moreover, previous studies showed that brain reorganization is not a static phenomenon but rather a dynamic process (Moxon et al., 2014). From the perspective view of mechanisms/pathophysiology, traumatic SCI is pathophysiologically divided into primary and secondary injuries and can be temporally divided into the acute (<48 h), subacute (48 h to 14 days), intermediate (14 days to 6 months), and chronic (>6 months) phases (Ahuja et al., 2017). In the chronic phase, the spinal cord lesion evolves alterations in remodeling of neural circuits (Kwon, 2004), and the increased FC plays an important role in the motor recovery of SCI at 6 months post‐injury (Hou et al., 2016). Interestingly, the sensorimotor functional network changes in different injury times, such as differences in early and chronic phases, have yet to be investigated.

In order to elucidate the FC changes between the sensorimotor cortex and other brain regions that might provide an adaptive compensatory method for functional recovery in SCI patients, we aimed to (1) investigate increased FC in the cortical connections between the sensorimotor cortex and other brain regions in individuals with complete SCI using resting‐state fMRI; (2) compare FC differences between early and late injury stages in SCI patients. We also hypothesized that compensatory FC increases between the sensorimotor cortex and other brain regions and these FC increases in different injury duration.

## 2 MATERIALS AND METHODS

### 2.1 Subjects

A total of 27 subjects with complete SCI (five females, 40.40 ± 11.50 years) and 30 healthy controls (HCs) (six females, 40.10 ± 10.55 years) were recruited in this study. The 27 SCI subjects were divided into two subgroups: 13 SCI subjects with time injury within 6 months (early‐stage) and 14 SCI subjects with time injury beyond 6 months (late‐stage).

The SCI subjects were evaluated based on the International Standards for the Neurological Classification of Spinal Cord Injury (ISNCSCI) published by the American Spinal Injury Association (ASIA) Impairment Scale (AIS) (Kirshblum, 2014). The neurological level of the injury is defined as the uppermost segment with neurologically intact motor and sensory scores. The SCI subjects were classified according to the neurological classification as AIS A (i.e., complete injury, no sensory or motor functions preserved in sacral segments), AIS B, C, or D (i.e., incomplete injury), or AIS E (i.e., no functional impairment). Moreover, the subjects were evaluated for motor and sensory function, including total motor score (i.e., upper + lower motor score) and total sensory score (i.e., light touch + pinprick score).

The inclusion criteria were as follows: SCI subjects were (1) aged 18–60 years; (2) evaluated as AIS A, that is, a complete injury without residual sensation in the sacral segments of the spinal cord; (3) traumatic SCI; (4) right‐handed. The exclusion criteria were as follows: (1) decreased cognition and inability to comprehend commands; (2) associated traumatic brain injury; (3) neuropathic pain, according to the visual analog scale; (4) severe contractures; (5) deformities of the skull; (6)psychiatric disorder; (7) contraindications to MRI; (8) inability to consent for procedures. Furthermore, the HCs had to be right‐handed, healthy without severe psychosomatic diseases and consent to the study. The SCI subjects and HCs were matched statistically for age and gender.

The study was carried out in accordance with the recommendations of The Medical Ethics Committee of China Rehabilitation Research Center (CRRC) (Beijing, China) (ref: 2017‐071‐1) with written informed consent from all subjects before the study, which was approved by the medical ethics committee of CRRC.

### 2.2 MRI scanning

All subjects were scanned on a 3T MRI scanner (Philips Ingenia, Best, The Netherlands) at the CRRC. For scanning, subjects were positioned supine on the gantry of the scanner with the head in a mid‐line location with a multichannel head coil and stabilized by clamps to reduce the motion‐related artifacts during scanning. During the resting‐state acquisitions, the subjects were instructed to close their eyes, relax, and stay awake without any concentrated thinking. The resting‐state fMRI scans were acquired using echo‐planar imaging (EPI) sequence with the following imaging parameters: slice thickness = 3.5 mm, time repetition (TR) = 2000 ms, time echo (TE) = 30 ms, flip angle (FA) = 90°, slice gap = 0.5, filed of view (FOV) = 230 × 230 mm^2^, matrix size = 80 × 80, voxel size = 3 × 3 × 3 mm^3^. In each subject, a high‐resolution 3D T1‐weighted anatomical image set covering the whole brain was collected. The scanning parameters were as follows: FOV = 256 × 256 mm, matrix size = 256 × 256, slice thickness = 0.87 mm, TR = 7600 ms, TE = 3.7 ms, FA = 8°, voxel size = 1 × 1 × 1 mm^3^.

### 2.3 Data preprocessing

The preprocessing of fMRI data was carried out using DPARSFA (V4.3) (http://rfmri.org/DPARSF) and SPM12 (V6906) (https://www.fil.ion.ucl.ac.uk/spm/software/spm12/). The first 10 time points were removed to allow the subjects to acclimatize with the scanning environment. Then, slice timing correction was performed. Head motion was corrected before normalizing the image to a 2‐mm isotropic BOLD EPI template in the Montreal Neurological Institute (MNI) 152 standard space. The image was resampled to 3‐mm isotropic voxels and spatially smoothed by a Gaussian kernel with 4 mm full‐width half‐maximum (FWHM). Subsequently, we removed the linear trend and nuisance covariates, including head motions, cerebral fluid, white matter, and the global signal. Finally, the signal time course was temporally filtered to keep the signals within 0.01–0.08 Hz.

### 2.4 Functional connectivity analysis (seed‐based analysis)

The data were processed using the in‐house Python software. The whole brain was parcellated into 84 Brodmann areas (BA) and 26 cerebellum regions from the Automated Anatomical Labeling (AAL) atlas in the MNI space (Tzourio‐Mazoyer et al., 2002). The BOLD time course within each brain area was averaged and Pearson's correlation coefficient was calculated for each pair of regions as the functional connectivity between these two nodes in the network.

We defined two sets of seed regions of interest (ROI). The first set of ROIs consisted of BA4 and BA6 from the motor regions of both hemispheres. The second set of ROIs consisted of BA1, 2, and 3, representing the somatosensory region.

### 2.5 Statistical analysis

Statistical analysis was performed using SPSS software, version 20.0 (SPSS Inc., Chicago, IL, USA). Continuous variables were tested using two‐tailed *t*‐tests, while gender differences were examined by chi‐square test (*p* < .05). For FC analysis, we utilized two‐sample *t*‐tests (*p* < .05) to identify increased FC associated with the above defined ROIs between SCI patients and HCs. We selected significantly increased FC between SCI patients and HCs. SCI subgroup analysis was performed by  further exploring the differences between SCI subgroups using two‐sample t‐tests (*p* < .05). Both increased and decreased FC was selected in subgroup analysis. The t‐test between subgroups served as a feature extraction procedure, followed by correlation analysis. Pearson's correlation analyses between significant FC obtained in the subgroup analysis and time since injury (injury duration) were performed to investigate the correlations between FC and clinical variables in SCI subjects; *p* < .05 indicated statistical significance.

## 3 RESULTS

The average age of the SCI group was 40.40 ± 11.50 years (five females) with injury duration from 1 month to 16 years; 13 SCI subjects (42.88 ± 9.95 years, two females) comprised the early‐stage group and 14 (38.10 ± 12.70 years, three females) constituted the late‐stage group. The average age of the HC group was 40.10 ± 10.55 years (six females). The chi‐square test did not show any significant differences in the gender distribution between SCI and HC groups and between early‐ and late‐stage groups. Table 1 summarizes the demographical information of SCI subjects.

**TABLE 1:** SCI patients information

ID	Sex	Age, years	Injury				Light touch (max 56 points)	Pinprick (max 56 points)	Motor score (max 50 points)	VAS
			Type	Duration, m	Level	AIS	L/R	L/R	L/R	
*Early stage*										
1	M	25	Vehicle accident	2	T11	A	38/40	36/37	25/25	0
2	M	54	Fall	4	T10	A	36/36	36/36	25/25	0
3	F	49	Fall	3	T12	A	42/42	40/40	27/27	0
4	M	51	Fall	2	C7	A	9/9	10/8	12/12	0
5	M	53	Crush by weight	5	T12	A	40/40	40/40	25/25	0
6	M	46	Fall	4	T10	A	36/37	36/37	25/25	0
7	F	30	Fall	4	T11	A	41/36	40/36	26/26	0
8	M	45	Crush by weight	5	C8	A	14/13	14/12	13/13	0
9	M	39	Fall	1	T10	A	35/35	34/34	25/25	0
10	M	50	Fall	1	T10	A	34/35	34/35	25/25	0
11	M	36	Crush by weight	3	T9	A	33/33	33/33	25/25	0
12	M	29	Crush by weight	1	T10	A	35/35	36/36	25/25	0
13	M	50	Fall	1	T10	A	35/35	35/35	25/25	0
*Late stage*										
14	M	52	Fall	7	T11	A	38/38	38/37	26/26	0
15	M	30	Crush by weight	13	T10	A	38/40	37/40	25/25	0
16	M	50	Crush by weight	81	T10	A	34/34	34/34	25/25	0
17	F	52	Vehicle accident	139	T11	A	36/36	36/36	25/25	0
18	M	60	Crush by weight	201	T9	A	32/32	33/33	25/25	0
19	M	26	Fall	55	T11	A	36/36	36/36	25/25	0
20	F	31	Vehicle accident	59	T5	A	27/26	27/26	25/25	0
21	F	37	Vehicle accident	105	T7	A	30/30	30/30	25/25	0
22	M	28	Crush by weight	56	T9	A	32/32	32/32	25/25	0
23	M	54	Vehicle accident	170	T10	A	36/36	36/36	25/25	0
24	M	26	Vehicle accident	15	T11	A	43/43	41/44	30/26	0
25	M	29	Fall	14	T11	A	39/39	39/39	25/25	0
26	M	23	Vehicle accident	42	T7	A	30/29	30/29	25/25	0
27	M	35	Crush by weight	38	C7	A	11/11	8/8	13/12	0

*Note*: Early stage: SCI patients with injury duration within 6 months; Late stage: SCI patients with injury duration beyond 6 months.

Abbreviations: F, female; M, male; VAS, visual analog scale.

**FIGURE 1:**
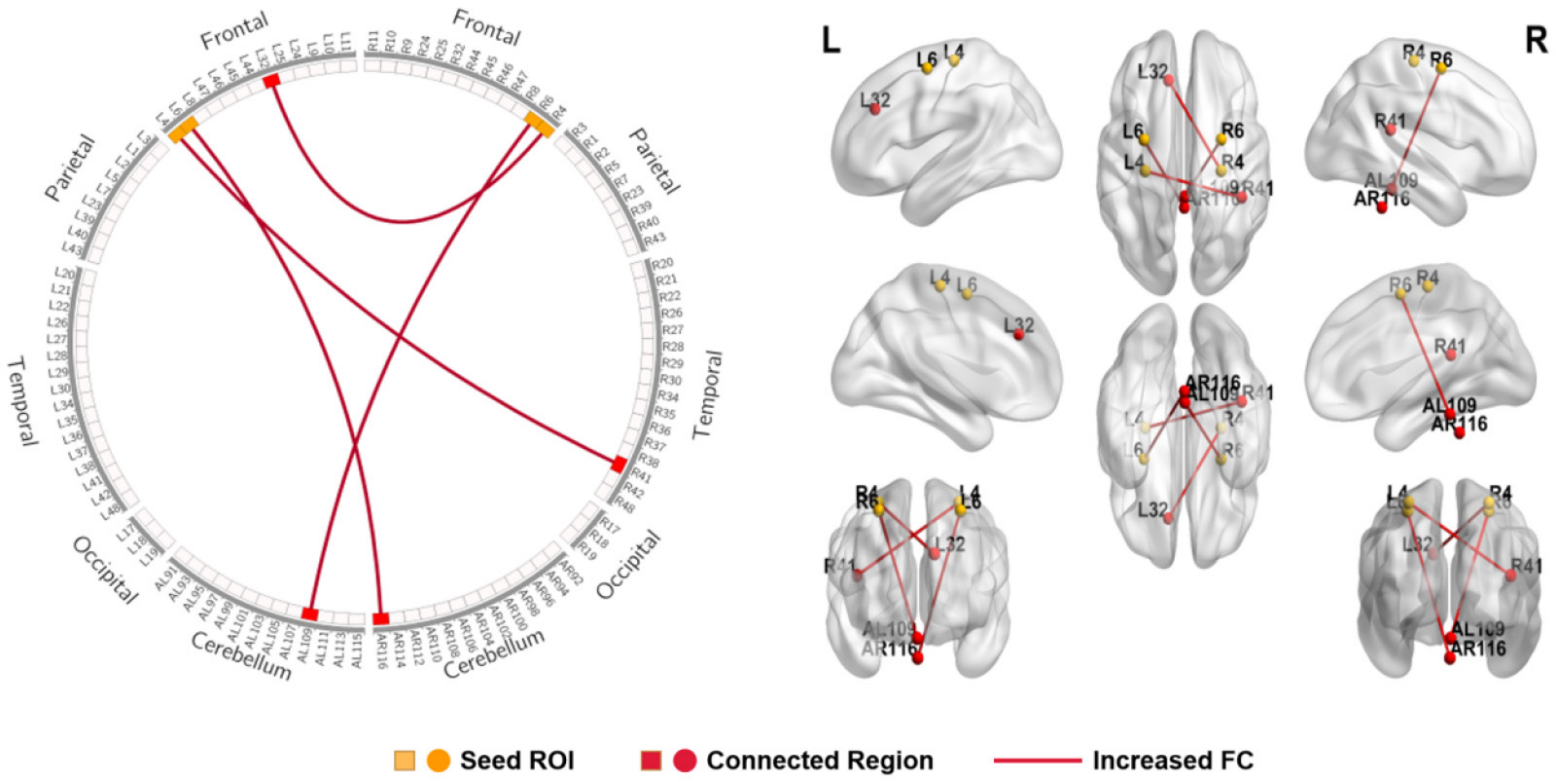
Schematic diagram and anatomic replicas show increased FC between motor cortices (BA4, BA6) and whole brain network in SCI patients compared with HCs. The yellow boxes and nodes represent the seed regions of FC. The red boxes and nodes represent the connected regions of FC. The red line denotes the increased FC in SCI patients compared with HCs. L, left; R, right.

**FIGURE 2:**
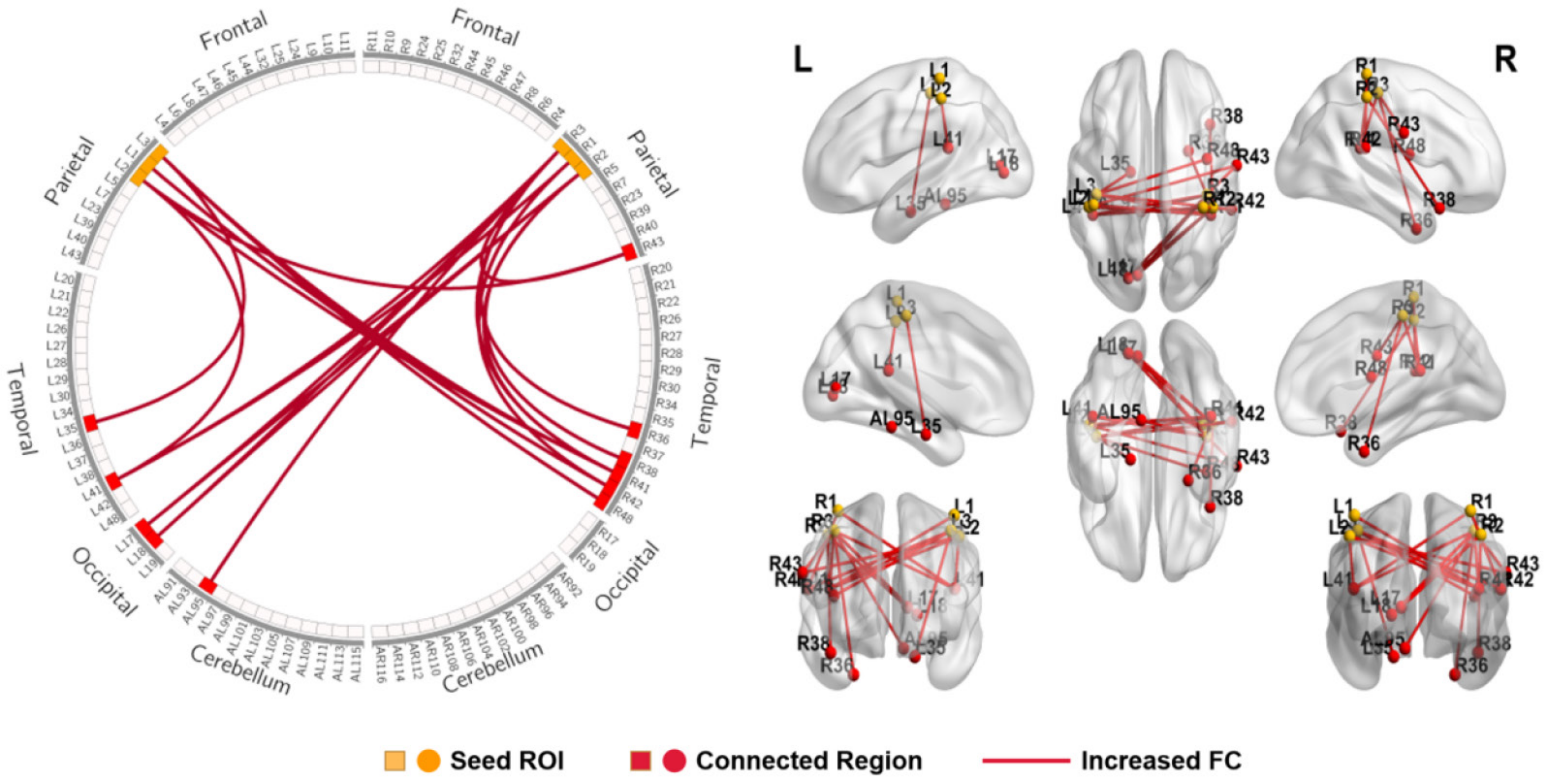
Schematic diagram and anatomic replicas show increased FC between somatosensory cortices (BA1, BA2, BA3) and whole brain network in SCI patients compared with HCs. The yellow boxes and nodes represent the seed regions of FC. The red boxes and nodes represent the connected regions of FC. The red line denotes the increased FC in SCI patients compared with HCs. L, left; R, right.

When motor cortices (BA4 and BA6) were selected as seed ROI, connectivity analysis showed an increase in FC between motor cortex and primary auditory cortex (right BA41), dorsal anterior cingulate cortex (ACC) (left BA32), and cerebellum (left AAL109, right AAL116) in the SCI group compared to HCs (Table 2, Figure 1). Moreover, an increase in FC between the somatosensory cortex and primary auditory and visual cortex was identified in the SCI group when somatosensory cortices (BA1, BA2, and BA3) were selected as seed ROI (Table 3, Figure 2).

**TABLE 2:** Increased FC between motor cortices (BA4, BA6) and whole brain network in SCI patients compared with HCs

Seed ROI	Connected region	*t*‐value	*p*‐value
L‐BA4	R‐BA41	2.083	.042
L‐BA6	R‐AAL116	3.034	.004
R‐BA4	L‐BA32	2.062	.044
R‐BA6	L‐AAL109	2.095	.041

Abbreviations: AAL, anatomical automatic labeling; AAL109,116, vermis; BA, Brodmann area; BA4, primary motor cortex; BA6, premotor cortex; BA32, dorsal anterior cingulate cortex (ACC); BA41, primary auditory cortex; L, left; R, right; *p* < .05.

**TABLE 3:** Increased FC between somatosensory cortices (BA1, BA2, BA3) and whole brain network in SCI patients compared with HCs

Seed ROI	Connected region	*t*‐value	*p*‐value
L‐BA1	R‐BA41	2.406	.020
L‐BA2	L‐BA41	2.067	.044
L‐BA2	R‐BA41	3.271	.002
L‐BA2	R‐BA43	2.335	.023
L‐BA3	L‐BA35	2.295	.026
L‐BA3	R‐BA41	3.339	.002
L‐BA3	R‐BA42	2.810	.007
L‐BA3	R‐BA48	2.124	.038
R‐BA1	L‐BA41	2.171	.034
R‐BA1	R‐BA42	2.807	.007
R‐BA2	L‐BA17	2.964	.005
R‐BA2	L‐BA18	2.402	.020
R‐BA2	R‐BA38	2.120	.039
R‐BA2	R‐BA41	2.092	.041
R‐BA3	L‐BA17	2.386	.021
R‐BA3	L‐BA41	2.255	.028
R‐BA3	L‐BA18	2.729	.009
R‐BA3	R‐BA36	2.028	.047
R‐BA3	R‐BA41	2.369	.021
R‐BA3	R‐BA43	2.162	.035
R‐BA3	L‐AAL95	2.729	.009

Abbreviations: AAL, anatomical automatic labeling; AAL95, vermis; BA, Brodmann area; BA1,2,3, primary somatosensory cortex; BA17, primary visual cortex (V1); BA18, secondary visual cortex (V2); BA35, perirhinal cortex; BA36, ectorhinal area; BA41,42, auditory cortex; BA43, primary gustatory cortex; BA48, retrosubicular area; *p* < .05.

**FIGURE 3:**
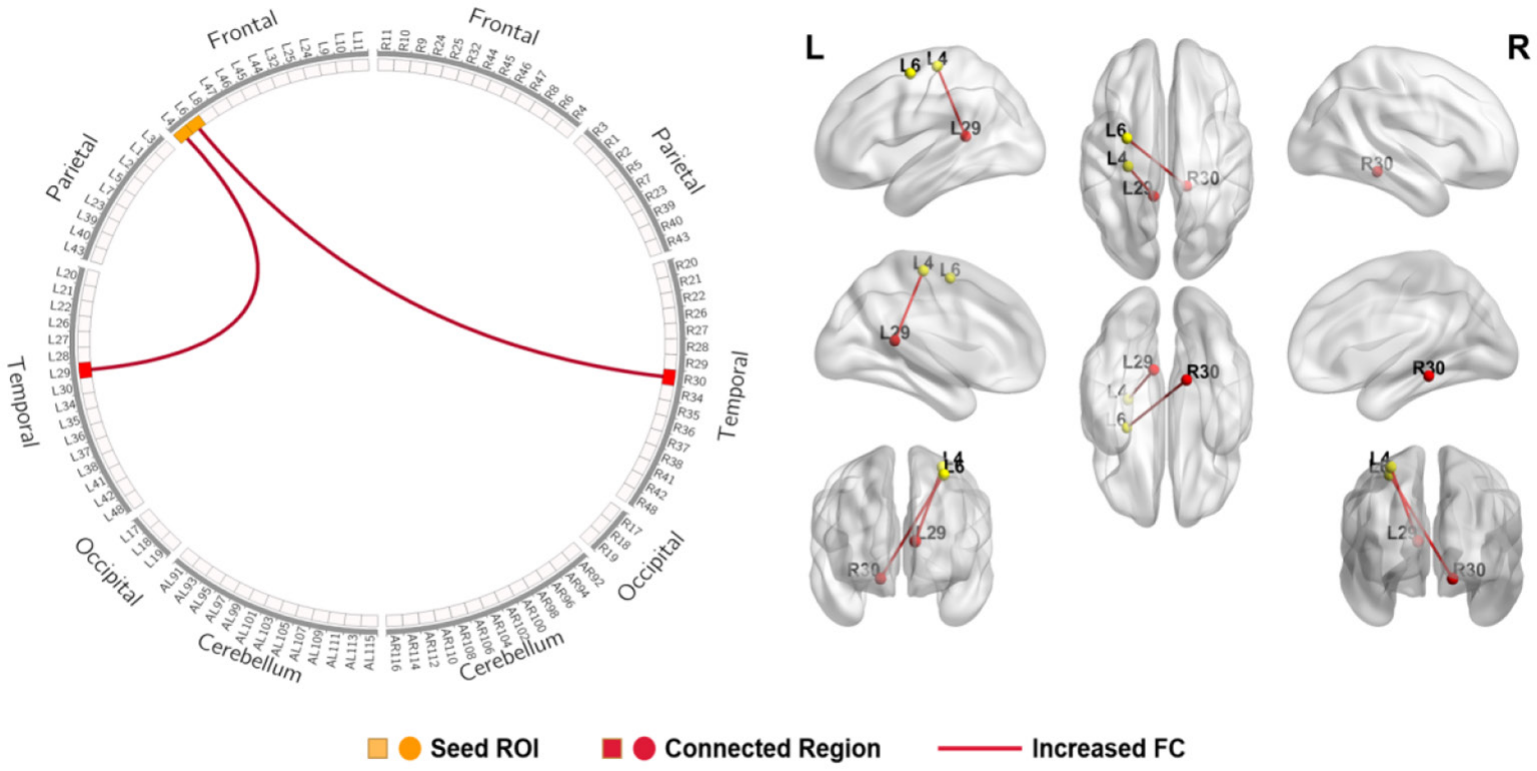
Schematic diagram and anatomic replicas show FC differences between motor cortices (BA4, BA6) and cingulate cortex (BA29, 30) between early‐stage group and late‐stage group. The yellow boxes and nodes represent the seed regions of FC. The red boxes and nodes represent the connected regions of FC. The red line denotes the increased FC in late‐ stage group relative to early‐stage group. L, left; R, right.

**FIGURE 4:**
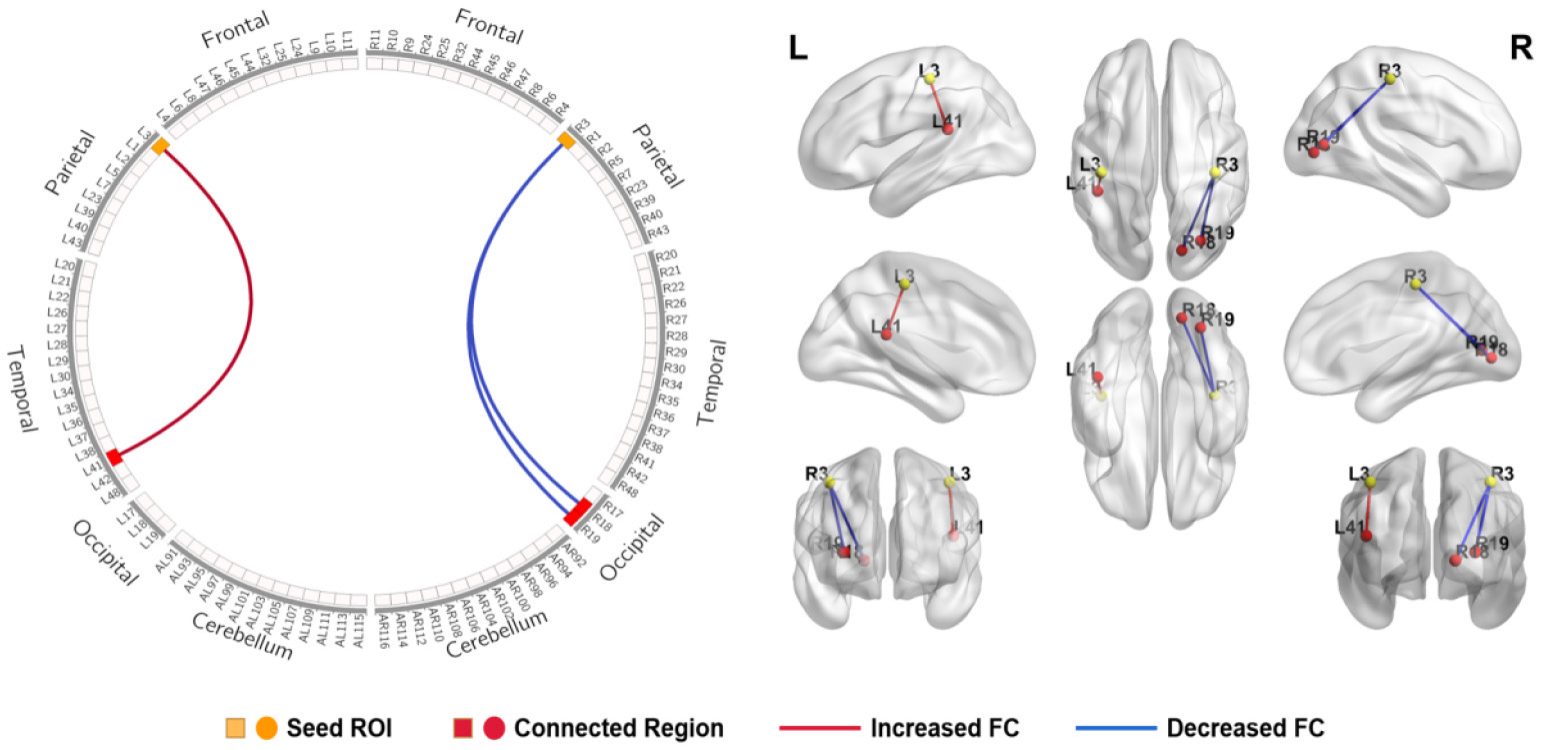
Schematic diagram and anatomic replicas show FC differences between somatosensory cortices (BA1, BA2, BA3) and visual, auditory regions between early‐stage group and late‐stage group. The yellow boxes and nodes represent the seed regions of FC. The red boxes and nodes represent the connected regions of FC. The red line denotes the increased FC, and the blue line denotes the decreased FC in late‐stage group relative to early‐stage group. L, left; R, right.

In the SCI subgroups analysis (late stage vs. early stage), compared to the early‐stage group, increased FC was found between the motor cortex and left retrosplenial cingulate cortex (BA29) and right cingulate cortex (BA30) in the late‐stage group when the seed ROI was located in the motor cortices (BA4 and BA6) (Table 4, Figure 3). Similar results were observed when the somatosensory cortex (BA1, BA2, and BA3) was selected as the seed ROI. The increased FC was observed between the somatosensory cortex and right primary visual cortex (BA18 and BA19) in the early‐stage group, while the increased FC was found between the somatosensory cortex and left primary auditory cortex (BA41) in the late‐stage group (Table 5, Figure 4).

**TABLE 4:** Differences of FC between motor cortex (BA4, BA6) and cingulate cortex (BA29, BA30) in SCI subgroups comparison

Seed ROI	Connected region	*t*‐value	*p*‐value
*Early stage* > *Late stage*			
None			
*Early stage* < *Late stage*			
L‐BA4	L‐BA29	−2.184	.039
L‐BA6	R‐BA30	−2.630	.014

*Note*: Early stage: SCI patients with injury duration within 6 months; Late stage: SCI patients with injury duration beyond 6 months; *p* < .05.

Abbreviations: BA29, retrosplenial cingulate cortex; BA30, part of cingulate cortex.

**TABLE 5:** Differences of FC between somatosensory (BA1, BA2, BA3) and visual, auditory regions in SCI subgroups comparison

Seed ROI	Connected region	*t*‐value	*p*‐value
*Early stage* > *Late stage*			
R‐BA3	R‐BA18	2.222	.036
R‐BA3	R‐BA19	2.291	.031
*Early stage* < *Late stage*			
L‐BA3	L‐BA41	−2.676	.013

*Note*: Early stage: SCI patients with injury duration within 6 months; Late stage: SCI patients with injury duration beyond 6 months; *p* < .05.

Abbreviations: BA18, secondary visual cortex (V2); BA19, associative visual cortex (V3,V4,V5); BA41, auditory cortex.

**FIGURE 5:**
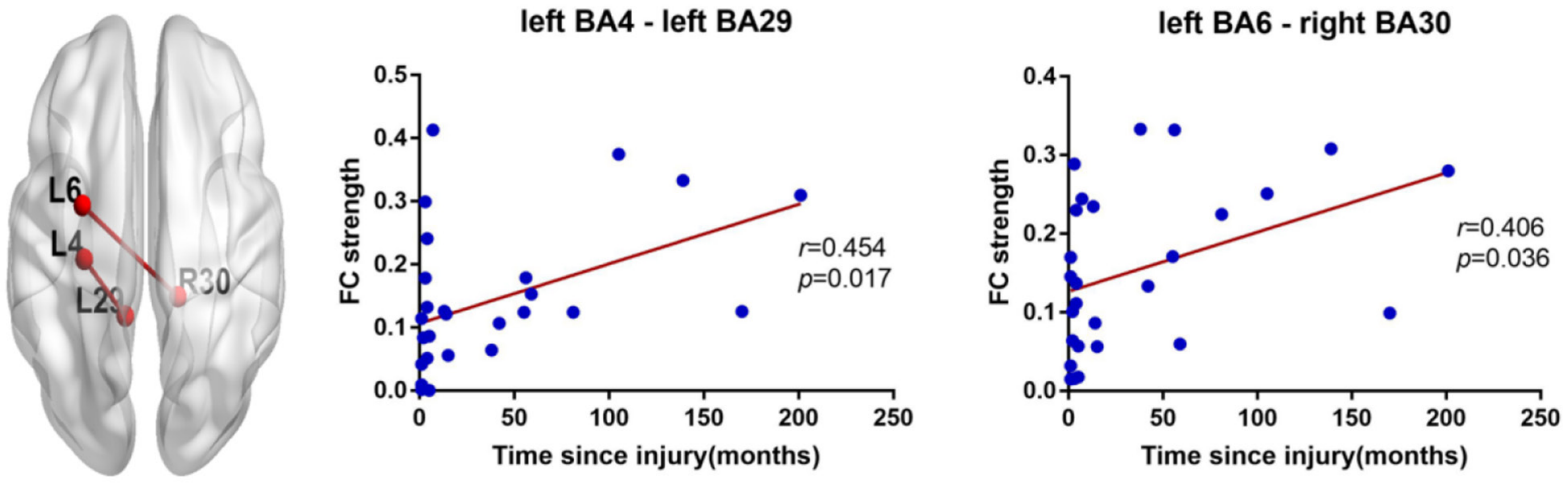
Correlation between FC strength and time since injury in SCI patients. The increased FC between left primary motor cortex (BA4) and left retrosplenial cingulate cortex (BA29) was positively correlated with the time since injury in SCI patients (*r* = 0.454, *p* = .017). The increased FC between left premotor cortex (BA6) and right part of cingulate cortex (BA30) was positively correlated with the time since injury in SCI patients (*r* = 0.406, *p* = .036).

Injury duration showed a positive correlation with FC values between left premotor cortex (BA6) and right cingulate cortex (BA30), controlling for age, gender, and nuisance covariates (*r* = 0.406, *p* = .036; Figure 5). Pearson's correlation also showed a positive correlation between injury duration and FC connecting left primary motor cortex (BA4) and left retrosplenial cingulate cortex (BA29) (*r* = 0.454, *p* = .017; Figure 5).

## 4 DISCUSSION

The present study investigated the increased sensorimotor network connectivity in complete SCI using resting‐state fMRI. Consistent with the hypothesis, SCI subjects showed an increase in FC between the sensorimotor cortex and cognitive, visual, and auditory cortices compared to HCs. The FC between motor cortex and cognitive cortex increased over time after injury. The FC between sensory cortex and visual cortex increased within 6 months after SCI, while the FC between sensory cortex and auditory cortex increased beyond 6 months. Overall, the findings demonstrated increased FC between the sensorimotor cortex and cognitive‐, visual‐, and auditory‐related regions following SCI, yet this connectivity changes occurred in different injury times.

### 4.1 FC differences between SCI subjects and HCs

The reorganization of the brain plays a vital role in the functional recovery in subjects with SCI (Wang et al., 2019). However, alternative therapies from high‐order interactions for the complete SCI subjects who have absolutely lost the sensorimotor function below the injury level are lacking. Functional neuroimaging studies in SCI subjects revealed functional alterations in the brain (Hou et al., 2014; Sabre et al., 2016; Wang et al., 2019). Compared to controls, SCI subjects showed increased activation in the sensorimotor cortex in both whole‐brain and ROI analyses in task fMRI studies (Cramer et al., 2007; Gustin et al., 2010; Henderson et al., 2011; Wrigley et al., 2009). Also, resting‐state fMRI studies indicated increased or decreased FC between sensorimotor cortices (Hawasli et al., 2018; Hou et al., 2014; Karunakaran et al., 2020; Min et al., 2015; Oni‐Orisan et al., 2016), which might be associated with clinical outcomes and have potential as responsive biomarkers of rehabilitation and treatment interventions (Grabher et al., 2015).

The current study found an increase in FC between motor cortex (BA4 and BA6) and left BA32 (dorsal anterior cingulate cortex), right BA41 (primary auditory cortex), left AAL109 (cerebellum), and right AAL116 (cerebellum) in complete SCI subjects compared to HCs. Our findings supported the conclusions from previous studies that reported increased FC in SCI subjects. Min et al. reported that SCI subjects had increased FC between the primary motor cortex and other motor areas, such as the supplementary motor area and basal ganglia (Min et al., 2015). Oni‐Orisan et al. demonstrated that the left postcentral had increased connectivity with the thalamus bilaterally in SCI subjects (Oni‐Orisan et al., 2016). Kaushal et al. observed an increased FC in a subnetwork of the sensorimotor cortex and cerebellum network in SCI (Kaushal et al., 2017a), and an increase of the network modularity (Kaushal et al., 2017b).

Furthermore, our results showed enhanced reciprocal communication of motor pathway and dorsal anterior cingulate cortex (ACC) (BA32), primary auditory cortex (BA41), cerebellum (AAL109 and AAL116). The dorsal ACC is located dorsal to the genu of the corpus callosum, which is considered as a cognitive, motor, and emotional structure (Heilbronner & Hayden, 2016; Shenhav et al., 2013, 2016). Several reports showed that dorsal ACC neurons are sensitive to both reward and movement directions, and the dorsal ACC promotes adjustments or changes in the action plans or abstract strategies (Shenhav et al., 2013). Thus, the increased FC between motor cortex and dorsal ACC indicated that dorsal ACC plays a compensatory role in motor control for the motor dysfunction in SCI subjects. The primary auditory cortex (BA41) receives information from the external auditory world (Murray et al., 2005). An increase in FC between the motor cortex and primary auditory cortex indicated that auditory information received from the outside environment after the active movement was lost in subjects with SCI. Regions AAL109 and AAL116 were located in the cerebellum; the tissue is crucial for coordinating voluntary movements (Fine et al., 2002) and cognitive processes (Buckner, 2013; Timmann & Daum, 2007). Thus, we speculated that increased FC in the cerebellum might contribute to the regulation of motor ability in SCI subjects. Similar findings were reported in several previous studies. Kaushal et al. reported that the FC of a subnetwork of the sensorimotor cortex and cerebellum network was increased in subjects with SCI (Kaushal et al., 2017b). Also, an increase in FC was noted between the cerebellum and both primary motor and sensory cortices in the study by Hawasli et al. (2018). Hou et al. (2014) showed an increased FC of M1 and cerebellum in incomplete SCI at ~12 weeks post‐injury. The paralyzed limbs could not execute motor function due to the blocked efferent information from the brain in complete SCI subjects. However, other cortices might facilitate recruitment of neural substrates to compensate for sensorimotor deficits in SCI, which implied that cognition‐ and auditory‐related functional training might play a vital role in the recovery of sensorimotor function after SCI.

Additionally, sensory pathways were damaged between the brain and periphery in subjects with SCI. How the brain reacts to the sensory dysfunction after SCI is yet to be elucidated. The current study showed an increase in FC between the sensory cortex and visual and auditory cortices in complete SCI subjects compared to HCs. This phenomenon might indicate that the visual and auditory cortices play a compensatory role in the sensory dysfunction of SCI subjects. In contrast to previous findings, our study found increased FC between the sensory cortex and visual cortex in SCI subjects. Hawasli et al. reported a decrease in FC between the visual cortex and the sensorimotor cortex (Hawasli et al., 2018). Chen et al. found that incomplete SCI subjects have decreased intra‐network FC in the medial vision network (mVN) (Chen et al., 2018). This might be associated with the differences in the severity, level, and duration (several days within 1 month) of injury in the SCI subjects. The subjects in this study were all complete SCI with injury time from subacute to chronic stages. Furthermore, the movement needed multisystem integration, such as neural control (sensorimotor cortex, visual cortex, auditory cortex, cerebellum, and spinal cord), musculoskeletal mechanics, and motor behavior (Scott, 2004). The neurons in the primary auditory cortex (BA41) are sensitive to orientation, luminance, or motion (Supèr, 2002). The current study first showed an increase in FC between the sensory cortex and primary auditory cortex, suggesting enhanced visual‐related sensory processing after somatosensory dysfunction in SCI subjects.

### 4.2 Differences in FC between SCI subgroups

The present study demonstrated increased FC between sensorimotor cortices and cognitive, visual, and auditory regions in SCI subjects compared to HCs. In order to investigate how brain FC changes over time after injury, we further compared the FC differences between SCI subgroups (early stage vs. late stage) and found increased FC between the motor cortex (BA4 and BA6) and cingulate cortex (BA29 and BA30) in the late‐stage group compared to the early‐stage group. The correlation analysis showed that increased FC between motor cortex and cingulate cortex had a positive correlation with time since injury in subjects with SCI. This finding indicated that the altered FC between the motor cortex and cognitive region is dynamic and varies through the recovery process after SCI. Although atrophy and degeneration are observed in the structural studies (Supèr, 2002), functional recovery might be improved. Similar to our findings, Hawasli et al. (2018) performed a seed‐based correlation mapping and established that connectivity changed over time after SCI. Cognition plays a crucial role in motor learning. Herein, we showed that high cognition might contribute to motor functional recovery over time following SCI. Thus, additional cognition training should be considered in SCI rehabilitation, such as motor image, motor relearning, and motor memory.

In addition, our study also demonstrated increased FC between sensory cortex and visual cortex within 6 months after SCI, while the FC between the sensory cortex and auditory cortex increased beyond 6 months. This finding suggested that sensory connectivity also changed over time after SCI. Vision and auditory might compensate for sensory dysfunction during the early‐ and late‐stage of SCI. This also reminds us that sensorimotor recovery is not static and we might need to change our rehabilitation plan over time to maximize recovery. In the first 6 months after injury, rehabilitation might be involved in the visual movement, while rehabilitation should be involved in the movement with respect to auditory functions beyond 6 months. For example, virtual reality (VR)‐augmented neurorehabilitation interventions might be helpful for motor recovery, which could provide multimodal sensory stimuli and environment feedback for the subjects, leading to brain reorganization (Villiger et al., 2015, 2017).

Nevertheless, the present study has several limitations. First, the study focused on the increased FC in SCI subjects compared to HCs; decreased FC might be explored in the subsequent studies. Second, the study only compared SCI subgroups with different injury durations; future longitudinal studies are necessary to explore the time course of functional reorganization in SCI subjects. Third, the current study focused on the FC reorganization pattern in the brain; multimodal imaging studies are necessary in the future.

## 5 CONCLUSION

In summary, the FC increased between the sensorimotor cortex and cognitive, visual, and auditory regions in SCI subjects. The FC increased over time after injury between the motor cortex and cognitive regions. The FC increased between the sensory cortex and visual cortex within 6 months after injury and between the sensory cortex and auditory cortex beyond 6 months. The brain FC changed at different injury stages after SCI.

### DECLARATION OF TRANSPARENCY

The authors, reviewers and editors affirm that in accordance to the policies set by the *Journal of Neuroscience Research*, this manuscript presents an accurate and transparent account of the study being reported and that all critical details describing the methods and results are present.

## ACKNOWLEDGMENTS

We thank all patients and healthy controls who participated in this study for their time and cooperation.

## AUTHOR CONTRIBUTIONS

Conceptualization, Y.P. and Y.G.; Methodology, Y.X.G, Y.G., Y.P., and W.B.D.; Investigation, Y.X.G, and Y.G.; Data Curation, Y.G. and J.J.L.; Project Administration, J.J.L.; Formal Analysis, Y.X.G, and Y.G.; Writing – Original Draft, Y.G.; Writing – Review & Editing, Y.X.G, Y.P., and W.B.D.; Supervision, Y.P. and J.J.L.; Funding Acquisition, Y.G. and J.J.L.

## CONFLICT OF INTEREST

The authors declare no potential conflict of interest.

## ETHICS APPROVAL

The study was approved by the medical ethics committee of CRRC (Beijing, China) (ref: 2017‐071‐1), and all subjects provided written informed consent.

### PEER REVIEW

The peer review history for this article is available at https://publons.com/publon/10.1002/jnr.25069.

## Supporting information

Transparent Science Questionnaire for Authors

## Data Availability

All the data supporting our findings are contained in the manuscript. The datasets used and/or analyzed in the current study are available from the corresponding author on reasonable request.

## References

[jnr25069-bib-0001] Ahuja, C. S. , Nori, S. , Tetreault, L. , Wilson, J. , Kwon, B. , Harrop, J. , Choi, D. , & Fehlings, M. G. (2017). Traumatic spinal cord injury—Repair and regeneration. Neurosurgery, 80, S9–S22. 10.1093/neuros/nyw080 28350947

[jnr25069-bib-0002] Azzarito, M. , Seif, M. , Kyathanahally, S. , Curt, A. , & Freund, P. (2020). Tracking the neurodegenerative gradient after spinal cord injury. Neuroimage: Clinical, 26, 102221. 10.1016/j.nicl.2020.102221 32145681 PMC7058923

[jnr25069-bib-0003] Buckner, R. L. (2013). The cerebellum and cognitive function: 25 years of insight from anatomy and neuroimaging. Neuron, 80, 807–815. 10.1016/j.neuron.2013.10.044 24183029

[jnr25069-bib-0004] Chen, Q. , Zheng, W. , Chen, X. , Li, X. , Wang, L. , Qin, W. , Li, K. , & Chen, N. (2018). Whether visual‐related structural and functional changes occur in brain of patients with acute incomplete cervical cord injury: A multimodal based MRI study. Neuroscience, 393, 284–294. 10.1016/j.neuroscience.2018.10.014 30326291

[jnr25069-bib-0005] Cramer, S. C. , Lastra, L. , Lacourse, M. G. , & Cohen, M. J. (2005). Brain motor system function after chronic, complete spinal cord injury. Brain, 128, 2941–2950. 10.1093/brain/awh648 16246866

[jnr25069-bib-0006] Cramer, S. C. , Orr, E. L. R. , Cohen, M. J. , & Lacourse, M. G. (2007). Effects of motor imagery training after chronic, complete spinal cord injury. Experimental Brain Research, 177, 233–242. 10.1007/s00221-006-0662-9 16944108

[jnr25069-bib-0007] Dietz, V. , & Curt, A. (2006). Neurological aspects of spinal‐cord repair: Promises and challenges. Lancet Neurology, 5, 688–694. 10.1016/S1474-4422(06)70522-1 16857574

[jnr25069-bib-0008] Fine, E. J. , Ionita, C. C. , & Lohr, L. (2002). The history of the development of the cerebellar examination. Seminars in Neurology, 22, 375–384. 12539058 10.1055/s-2002-36759

[jnr25069-bib-0009] Friston, K. J. (2011). Functional and effective connectivity: A review. Brain Connectivity, 1, 13–36. 10.1089/brain.2011.0008 22432952

[jnr25069-bib-0010] Grabher, P. , Callaghan, M. F. , Ashburner, J. , Weiskopf, N. , Thompson, A. J. , Curt, A. , & Freund, P. (2015). Tracking sensory system atrophy and outcome prediction in spinal cord injury. Annals of Neurology, 78, 751–761. 10.1002/ana.24508 26290444 PMC4737098

[jnr25069-bib-0011] Gustin, S. M. , Wrigley, P. J. , Siddall, P. J. , & Henderson, L. A. (2010). Brain anatomy changes associated with persistent neuropathic pain following spinal cord injury. Cerebral Cortex, 20, 1409–1419. 10.1093/cercor/bhp205 19815621

[jnr25069-bib-0012] Hawasli, A. H. , Rutlin, J. , Roland, J. L. , Murphy, R. K. J. , Song, S. , Leuthardt, E. C. , Shimony, J. S. , & Ray, W. Z. (2018). Spinal cord injury disrupts resting‐state networks in the human brain. Journal of Neurotrauma, 35, 864–873. 10.1089/neu.2017.5212 29179629 PMC5863102

[jnr25069-bib-0013] Heilbronner, S. R. , & Hayden, B. Y. (2016). Dorsal anterior cingulate cortex: A bottom‐up view. Annual Review of Neuroscience, 39, 149–170. 10.1146/annurev-neuro-070815-013952 PMC5512175 27090954

[jnr25069-bib-0014] Henderson, L. A. , Gustin, S. M. , Macey, P. M. , Wrigley, P. J. , & Siddall, P. J. (2011). Functional reorganization of the brain in humans following spinal cord injury: Evidence for underlying changes in cortical anatomy. Journal of Neuroscience, 31, 2630–2637. 10.1523/JNEUROSCI.2717-10.2011 21325531 PMC6623700

[jnr25069-bib-0015] Hou, J. M. , Sun, T. S. , Xiang, Z. M. , Zhang, J. Z. , Zhang, Z. C. , Zhao, M. , Zhong, J. F. , Liu, J. , Zhang, H. , Liu, H. L. , Yan, R. B. , & Li, H. T. (2014). Alterations of resting‐state regional and network‐level neural function after acute spinal cord injury. Neuroscience, 277, 446–454. 10.1016/j.neuroscience.2014.07.045 25086312

[jnr25069-bib-0016] Hou, J. , Xiang, Z. , Yan, R. , Zhao, M. , Wu, Y. , Zhong, J. , Guo, L. , Li, H. , Wang, J. , Wu, J. , Sun, T. , & Liu, H. (2016). Motor recovery at 6 months after admission is related to structural and functional reorganization of the spine and brain in patients with spinal cord injury. Human Brain Mapping, 37, 2195–2209. 10.1002/hbm.23163 26936834 PMC6867385

[jnr25069-bib-0017] Jutzeler, C. R. , Freund, P. , Huber, E. , Curt, A. , & Kramer, J. L. K. (2015). Neuropathic pain and functional reorganization in the primary sensorimotor cortex after spinal cord injury. Journal of Pain, 16, 1256–1267. 10.1016/j.jpain.2015.08.008 26392031

[jnr25069-bib-0018] Jutzeler, C. R. , Huber, E. , Callaghan, M. F. , Luechinger, R. , Curt, A. , Kramer, J. L. K. , & Freund, P. (2016). Association of pain and CNS structural changes after spinal cord injury. Scientific Reports, 6, 18534. 10.1038/srep18534 26732942 PMC4702091

[jnr25069-bib-0019] Karunakaran, K. D. , Yuan, R. , He, J. , Zhao, J. , Cui, J. , Zang, Y. , Zhang, Z. , Alvarez, T. L. , & Biswal, B. B. (2020). Resting‐state functional connectivity of the thalamus in complete spinal cord injury. Neurorehabilitation and Neural Repair, 34, 122–133. 10.1177/1545968319893299 31904298

[jnr25069-bib-0021] Kaushal, M. , Oni‐Orisan, A. , Chen, G. , Li, W. , Leschke, J. , Ward, B. D. , Kalinosky, B. , Budde, M. D. , Schmit, B. D. , Li, S. J. , Muqeet, V. , & Kurpad, S. N. (2017a). Large‐scale network analysis of whole‐brain resting‐state functional connectivity in spinal cord injury: A comparative study. Brain Connectivity, 7, 413–423. 10.1089/brain.2016.0468 28657334

[jnr25069-bib-0020] Kaushal, M. , Oni‐Orisan, A. , Chen, G. , Li, W. , Leschke, J. , Ward, B. D. , Kalinosky, B. , Budde, M. D. , Schmit, B. D. , Li, S. J. , Muqeet, V. , & Kurpad, S. N. (2017b). Evaluation of whole‐brain resting‐state functional connectivity in spinal cord injury: A large‐scale network analysis using network‐based statistic. Journal of Neurotrauma, 34, 1278–1282. 10.1089/neu.2016.4649 27937140

[jnr25069-bib-0022] Kirshblum, S. , & III Waring , W. (2014). Updates for the international standards for neurological classification of spinal cord injury. Physical Medicine and Rehabilitation Clinics of North America, 25, 505–517. 25064785 10.1016/j.pmr.2014.04.001

[jnr25069-bib-0023] Kupers, R. , & Ptito, M. (2014). Compensatory plasticity and cross‐modal reorganization following early visual deprivation. Neuroscience and Biobehavioral Reviews, 41, 36–52. 10.1016/j.neubiorev.2013.08.001 23954750

[jnr25069-bib-1000] Kwon, B. (2004). Pathophysiology and pharmacologic treatment of acute spinal cord injury. The Spine Journal, 4, 451–464. 10.1016/j.spinee.2003.07.007 15246307

[jnr25069-bib-0024] Min, Y. , Park, J. W. , Jin, S. U. , Jang, K. E. , Nam, H. U. , Lee, Y. , Jung, T. D. , & Chang, Y. (2015). Alteration of resting‐state brain sensorimotor connectivity following spinal cord injury: A resting‐state functional magnetic resonance imaging study. Journal of Neurotrauma, 32, 1422–1427. 10.1089/neu.2014.3661 25945389

[jnr25069-bib-0025] Moxon, K. A. , Oliviero, A. , Aguilar, J. , & Foffani, G. (2014). Cortical reorganization after spinal cord injury: Always for good? Neuroscience, 283, 78–94. 10.1016/j.neuroscience.2014.06.056 24997269 PMC4556279

[jnr25069-bib-0026] Murray, M. M. , Molholm, S. , Michel, C. M. , Heslenfeld, D. J. , Ritter, W. , Javitt, D. C. , Schroeder, C. E. , & Foxe, J. J. (2005). Grabbing your ear: Rapid auditory‐somatosensory multisensory interactions in low‐level sensory cortices are not constrained by stimulus alignment. Cerebral Cortex, 15, 963–974. 10.1093/cercor/bhh197 15537674

[jnr25069-bib-0027] Nardone, R. , Höller, Y. , Brigo, F. , Seidl, M. , Christova, M. , Bergmann, J. , Golaszewski, S. , & Trinka, E. (2013). Functional brain reorganization after spinal cord injury: Systematic review of animal and human studies. Brain Research, 1504, 58–73. 10.1016/j.brainres.2012.12.034 23396112

[jnr25069-bib-0028] Oni‐Orisan, A. , Kaushal, M. , Li, W. , Leschke, J. , Ward, B. D. , Vedantam, A. , Kalinosky, B. , Budde, M. D. , Schmit, B. D. , Li, S. J. , Muqeet, V. , & Kurpad, S. N. (2016). Alterations in cortical sensorimotor connectivity following complete cervical spinal cord injury: A prospective resting‐state fMRI study. PLoS ONE, 11, e0150351. 10.1371/journal.pone.0150351 26954693 PMC4783046

[jnr25069-bib-0029] Osinski, T. , Acapo, S. , Bensmail, D. , Bouhassira, D. , & Martinez, V. (2020). Central nervous system reorganization and pain after spinal cord injury: Possible targets for physical therapy—A systematic review of neuroimaging studies. Physical Therapy, 100, 946–962. 10.1093/ptj/pzaa043 32201890

[jnr25069-bib-0030] Sabre, L. , Tomberg, T. , Kõrv, J. , Kepler, J. , Kepler, K. , Linnamägi, Ü. , & Asser, T. (2016). Brain activation in the chronic phase of traumatic spinal cord injury. Spinal Cord, 54, 65–68. 10.1038/sc.2015.158 26391190

[jnr25069-bib-0031] Scott, S. H. (2004). Optimal feedback control and the neural basis of volitional motor control. Nature Reviews Neuroscience, 5, 532–545. 10.1038/nrn1427 15208695

[jnr25069-bib-0032] Shenhav, A. , Botvinick, M. M. , & Cohen, J. D. (2013). The expected value of control: An integrative theory of anterior cingulate cortex function. Neuron, 79, 217–240. 10.1016/j.neuron.2013.07.007 23889930 PMC3767969

[jnr25069-bib-0033] Shenhav, A. , Cohen, J. D. , & Botvinick, M. M. (2016). Dorsal anterior cingulate cortex and the value of control. Nature Neuroscience, 19, 1280–1285. 10.1038/nn.4382 27669989

[jnr25069-bib-0034] Supèr, H. (2002). Cognitive processing in the primary visual cortex: From perception to memory. Reviews in the Neurosciences, 13, 287–298. 12542258 10.1515/revneuro.2002.13.4.287

[jnr25069-bib-0035] Timmann, D. , & Daum, I. (2007). Cerebellar contributions to cognitive functions: A progress report after two decades of research. The Cerebellum, 6, 159–162. 10.1080/14734220701496448 17786810

[jnr25069-bib-0036] Tzourio‐Mazoyer, N. , Landeau, B. , Papathanassiou, D. , Crivello, F. , Etard, O. , Delcroix, N. , Mazoyer, B. , & Joliot, M. (2002). Automated anatomical labeling of activations in SPM using a macroscopic anatomical parcellation of the MNI MRI single‐subject brain. Neuroimage, 15, 273–289. 10.1006/nimg.2001.0978 11771995

[jnr25069-bib-0037] Villiger, M. , Grabher, P. , Hepp‐Reymond, M. , Kiper, D. , Curt, A. , Bolliger, M. , Hotz‐Boendermaker, S. , Kollias, S. , Eng, K. , & Freund, P. (2015). Relationship between structural brainstem and brain plasticity and lower‐limb training in spinal cord injury: A longitudinal pilot study. Frontiers in Human Neuroscience, 9, 254. 10.3389/fnhum.2015.00254 25999842 PMC4420931

[jnr25069-bib-0038] Villiger, M. , Liviero, J. , Awai, L. , Stoop, R. , Pyk, P. , Clijsen, R. , Curt, A. , Eng, K. , & Bolliger, M. (2017). Home‐based virtual reality‐augmented training improves lower limb muscle strength, balance, and functional mobility following chronic incomplete spinal cord injury. Frontiers in Neurology, 8, 635. 10.3389/fneur.2017.00635 29234302 PMC5712347

[jnr25069-bib-0039] Wang, W. , Xie, W. , Zhang, Q. , Liu, L. , Liu, J. , Zhou, S. , Shi, J. , Chen, J. , & Ning, B. (2019). Reorganization of the brain in spinal cord injury: A meta‐analysis of functional MRI studies. Neuroradiology, 61, 1309–1318. 10.1007/s00234-019-02272-3 31420686

[jnr25069-bib-0040] Wrigley, P. J. , Press, S. R. , Gustin, S. M. , Macefield, V. G. , Gandevia, S. C. , Cousins, M. J. , Middleton, J. W. , Henderson, L. A. , & Siddall, P. J. (2009). Neuropathic pain and primary somatosensory cortex reorganization following spinal cord injury. Pain, 141, 52–59. 10.1016/j.pain.2008.10.007 19027233

[jnr25069-bib-0041] Yoon, E. J. , Kim, Y. K. , Shin, H. I. , Lee, Y. , & Kim, S. E. (2013). Cortical and white matter alterations in patients with neuropathic pain after spinal cord injury. Brain Research, 1540, 64–73. 10.1016/j.brainres.2013.10.007 24125807

[jnr25069-bib-0042] Zhu, L. , Wu, G. , Zhou, X. , Li, J. , Wen, Z. , & Lin, F. (2015). Altered spontaneous brain activity in patients with acute spinal cord injury revealed by resting‐state functional MRI. PLoS ONE, 10, e0118816. 10.1371/journal.pone.0118816 25768010 PMC4359126

